# Iterative ensemble feature selection for multiclass classification of imbalanced microarray data

**DOI:** 10.1186/s40709-016-0045-8

**Published:** 2016-07-04

**Authors:** Junshan Yang, Jiarui Zhou, Zexuan Zhu, Xiaoliang Ma, Zhen Ji

**Affiliations:** College of Engineering and Information, Shenzhen University, Shenzhen, People’s Republic of China; School of Computer Science and Technology, Harbin Institute of Technology Shenzhen Graduate School, Shenzhen, People’s Republic of China; College of Computer Science and Software Engineering, Shenzhen University, Shenzhen, People’s Republic of China

## Abstract

**Background:**

Microarray technology allows biologists to monitor expression levels of thousands of genes among various tumor tissues. Identifying relevant genes for sample classification of various tumor types is beneficial to clinical studies. One of the most widely used classification strategies for multiclass classification data is the One-Versus-All (OVA) schema that divides the original problem into multiple binary classification of one class against the rest. Nevertheless, multiclass microarray data tend to suffer from imbalanced class distribution between majority and minority classes, which inevitably deteriorates the performance of the OVA classification.

**Results:**

In this study, we propose a novel iterative ensemble feature selection (IEFS) framework for multiclass classification of imbalanced microarray data. In particular, filter feature selection and balanced sampling are performed iteratively and alternatively to boost the performance of each binary classification in the OVA schema. The proposed framework is tested and compared with other representative state-of-the-art filter feature selection methods using six benchmark multiclass microarray data sets. The experimental results show that IEFS framework provides superior or comparable performance to the other methods in terms of both classification accuracy and area under receiver operating characteristic curve. The more number of classes the data have, the better performance of IEFS framework achieves.

**Conclusions:**

Balanced sampling and feature selection together work well in improving the performance of multiclass classification of imbalanced microarray data. The IEFS framework is readily applicable to other biological data analysis tasks facing the same problem.

## Background

Microarray gene expression data are widely used for cancer clinical studies [1, 2].The identification of relevant genes to cancers is a common biological challenge [3]. It is crucial to explore a list of high-potential biomarkers and signature candidates that are strongly associated with the disease among a large number of simultaneously observed genes [4]. From a machine learning perspective, gene selection is regarded as feature selection to the candidate genes that can be used to distinguish the classes of sample tissues.

Multiclass cancer prediction based on gene selection has attracted increasing research interest [5–8]. For instance, Li et al. [9] compared different feature selection and multiclass classification methods for gene expression data. The paper indicated that multiclass classification problem is much more difficult than the binary one for gene expression data. By comparing several filter feature selection methods and representative classifiers including naive Bayes, k-nearest neighbor (KNN), and support vector machine (SVM), they also suggested that the classification accuracy degrades rapidly as the number of classes increase. Kim-Anh et al. [10] developed a One-Verse-One schema based optimal feature weighting approach using classification-and-regression tree and SVM classifiers. Zhou et al. [7] extended the support vector machine—recursive feature elimination (SVM-RFE) [11] to solve the multiclass gene selection problem based on different frameworks of multiclass SVMs, and improved the classification accuracy. Yeung et al. [12] utilized the Bayesian model averaging method for gene selection, which was reported to be applicable to microarray data sets with any number of classes. It is capable of obtaining high accuracy with only a small number of selected genes, and meanwhile providing posterior probabilities for the predictions. To alleviate the siren-pitfall problem, Rajapakse et al. [8] proposed a novel algorithm to decompose multiclass ranking statistics into class-specific statistics, and use Pareto-front analysis for the selection of genes. Experiments showed that a significant improvement in classification performance and redundancy reduction among the top-ranked genes was achieved.

The aforementioned methods have achieved success in multiclass microarray data, however, the inherent imbalanced nature of multiclass microarray data, i.e., some minority classes may have relatively small number of samples compared to other classes (denoted as majority classes), still pose major challenges to gene selection methods. In this study, we propose an iterative ensemble feature selection (IEFS) framework based on the One-Versus-All (OVA) classification schema [13] to improve the classification performance in terms of both classification accuracy and area under receiver operating characteristic curve (AUC). OVA schema is a widely used ensemble solution for solving multiclass problems. In each binary sub-classification of OVA schema, samples of the majority class outnumber those from the minority class [14–17]. Therefore, a binary classifier would obtain good overall accuracy on majority class but not the minority class. The informative genes beneficial to separate the minority class are overwhelmed by those that are discriminating in the majority class, due to the lack of samples in minority class. Known as siren-pitfall, this problem has not yet been well addressed in multiclass classification of microarray data [18]. In this paper, we use a sampling method prior to gene selection in binary classification to solve this problem caused by imbalanced data distribution.

Data sampling is one of the most widely used approaches to address imbalanced classification problem [19]. It turns an imbalanced distribution data into a balanced/optimal distribution one, wherein undersampling and oversampling as the two representative approaches have been thoroughly studied [19]. Undersampling removes samples from the majority class to match the minority class. In contrast, oversampling duplicates samples from the minority class to match the size of majority class [20].

The IEFS framework is tested on six benchmark multiclass microarray data sets and the experimental results show that the framework significantly improves the prediction accuracy of both minority and majority classes.

## Results and discussion

### Microarray data sets

To validate the effectiveness of IEFS framework, six multiclass benchmark microarray data sets shown in Table 1 are used in the experiments. The GCM data consists of 190 tumor samples of 14 commonly seen tumor types. Lung data contains 186 lung tumor samples and 17 normal tissues. The 186 lung tumor samples include 139 adenocarcinomas resected from lung and 47 tumor samples from the other 3 tumor types. In the acute lymphoblastic leukemia (ALL) data set, target and classes cover 7 subgroups from the treatment of pediatric ALL. The ALL-AML-4 data set comes with 4 types of tumors including AML-Bone Marrow, ALL-Peripheral Blood, ALL B cell, and T-Cell. The ALL-AML-3 data set has 3 types of tumors including AML, ALL B-Cell, and ALL T-Cell with 7129 samples. Finally, the Thyroid data set profiles from the human thyroid origin with four thyroid tissue types, i.e., papillary carcinoma, follicular carcinoma, follicular adenoma, and normal thyroid. All these data sets are characterized with only a small number of samples but thousands of features. The number of classes in all data sets range from 3 to 14.

**Table 1:** Summary of microarray data sets

Name	#Features	#Samples	#Samples in each class	#Classes	Source
GCM	14,122	190	11 10 11 11 22 11 10 10 30 11 11 11 11 20	14	[14]
Lung	12,600	203	139 17 6 21 20	5	[15]
ALL	12,558	327	15 27 64 20 43 79 79	7	[16]
ALL-AML-4	7129	72	38 9 21 4	4	[17]
ALL-AML-3	7129	72	38 9 25	3	[17]
Thyroid	2000	168	58 28 40 42	4	[21]

### Experimental design

In the experiment, we investigate the combinations of two sampling methods, i.e., oversampling and undersampling, and three filter feature selection methods in IEFS framework. The filter feature selection methods include one ranking method and two space search methods [22]. The ranking method measures the relevance between features and the class label vector based on mutual information [23]. The two space search methods include fast correlation-based filter selection (FCBF) [24] and minimum redundancy maximum relevance feature selection (mRMR) [25]. FCBF identifies relevant features as well as redundancy among them based on symmetric uncertainty. The mRMR penalises a feature’s relevancy by its redundance in the presence of the other selected features. The relevance and redundancy are measured using correlation between features based on mutual information.

In the IEFS framework, undersampling or oversampling technology is applied to correct the sample distribution skewness before feature selection. Particularly, the random undersampling [26] and the synthetic minority oversampling technique (SMOTE) [27] are used. The sampling and feature selection are performed iteratively and alternatively until a satisfactory performance is obtained.

The classification performance of the selected feature subset obtained by IEFS framework is evaluated using both KNN and SVM. KNN and SVM classifiers are sensitive to the imbalanced class distribution [28, 29]. Their performance on the imbalanced data sets can easily be affected without sample distribution skewness correction. IEFS framework is expected to improve the performance of KNN and SVM.

Most classifiers obtain good overall classification accuracy on the whole data but a poor accuracy on the minority classes [30]. When applied on imbalanced data, a good classifier should perform well on minority classes even at the expense of performance on the majority classes. AUC measures the sensitivity and specificity that are defined as the proportions of samples that are correctly classified in the positive and the negative classes, respectively. Therefore, the metric of AUC is better than classification accuracy to evaluate classifier performance on minority class [31]. In addition to classification accuracy, the classification performance on AUC is also reported.

In our empirical studies, the number of selected features in filter ranking method is increased from 5 to 100 with internal 5. The performance of using all features is introduced as the baseline performance. The number of the nearest neighbors used in oversampling method is set to 5. For the controlled size of selected feature with filter ranking method, the step *T* of sample balance and feature selection is set to 1 and 4, respectively. Because FCBF is capable of deciding the number of selected features itself, the step *T* of sample balance and feature selection in IEFS with FCBF is set to 1. Consistently, the step *T* of selected feature subset with mRMR is set to 1. The classification accuracies on data sets Lung, ALL-AML-3 and ALL-AML-4 are evaluated with threefold stratified cross-validation [31] as the sizes of some classes are smaller than 10. The classification accuracies on the other three data sets, i.e., GCM, ALL and Thyroid are evaluated using tenfold stratified cross-validation [32]. All experiments are conducted in the WEKA environment [33]. The other parameters for FCBF, mRMR and the classifiers [KNN (K = 3) and SVM] are used with default settings in WEKA.

### Experimental results

**Fig. 1:**
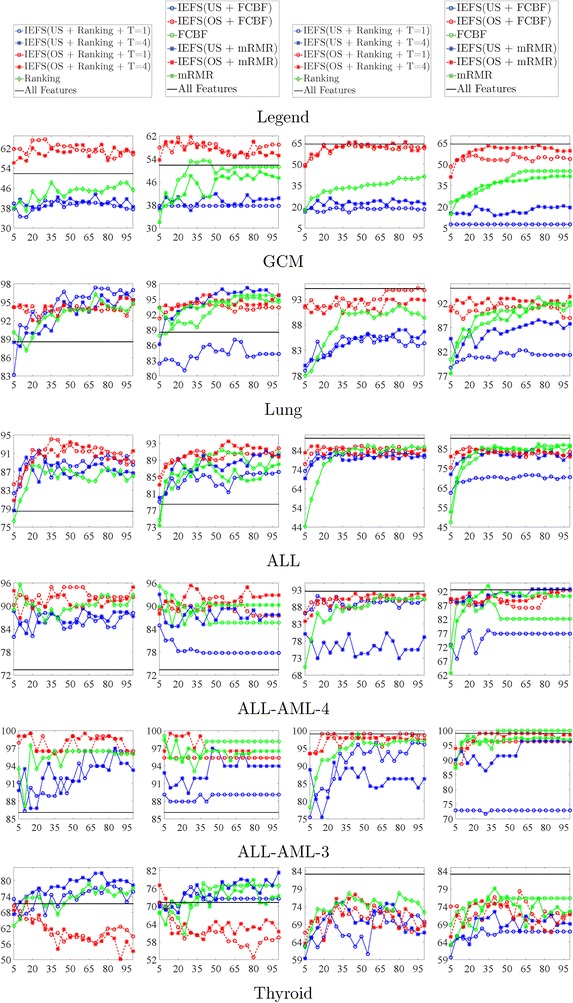
Comparison of classification accuracy using KNN and SVM. The* y-axis* indicates the classification accuracy (in percentage, %). The *x-axis* indicates the number of selected gene signatures. In the legend, “Undersampling” is abbreviated to “US” and “Oversampling” is abbreviated to “OS”. The *first* and the *third column* are the experiments using KNN. The *second* and the *fourth column* are the experiments using SVM

Figure 1 reports the classification accuracies of IEFS framework and other compared feature selection methods (Filter Ranking, FCBF and mRMR) using both KNN and SVM. The results on GCM data show that the classification accuracy of IEFS framework with oversampling (SMOTE) significantly outperforms other counterpart methods at various signature sizes. However, IEFS framework with undersampling is worse than the compared methods due to the extreme lack of training data. On data sets Lung, ALL, ALL-AML-3 and ALL-AML-4, the performance obtained by IEFS framework with oversampling (*T* = 1 and *T* = 4) is slightly better than the other methods. It is observed that the more classes a data set has, the better performance the proposed framework can obtain. This is because IEFS with oversampling (SMOTE) can correct the imbalanced sample distribution, and overcome the caused problem in single binary classification of the OVA schema. Meanwhile, oversampling can increase the number of training data that are beneficial to train a stronger binary classifier. IEFS framework outperforms other counterpart feature selection methods on most of the data sets by a decent margin of at least 10 selected features. This is very useful for biological data analysis, because biologists prefer fewer target genes so that they can focus more on those potential biomarkers. However, on Thyroid data, from Fig. 1 shows that the accuracy of IEFS framework with oversampling degrades sharply as the number of selected features increases. On the contrary, the accuracy of IEFS framework with undersampling increases obviously, especially with the ranking feature selection and KNN classifier.

**Fig. 2:**
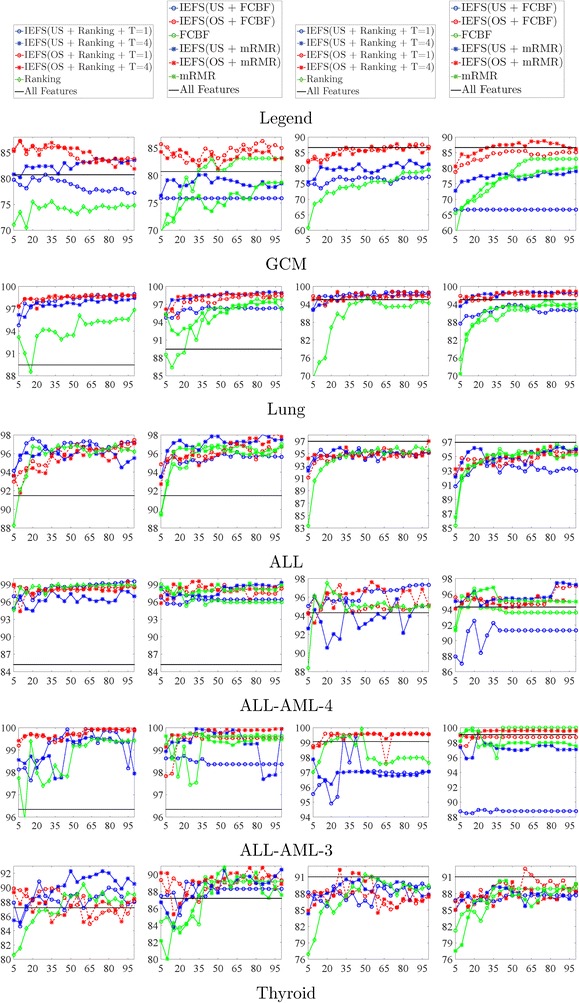
Comparison of AUC using KNN and SVM. The* y-axis* indicates AUC (in percentage, %). The* x-axis* indicates the number of selected gene signatures. In the legend, “Undersampling” is abbreviated to “US” and “Oversampling” is abbreviated to “OS”. The *first* and the *third column* are the experiments using KNN. The *second* and the *fourth column* are the experiments using SVM

The classification performance on AUC is reported in Fig. 2. The results on the GCM data show that IEFS framework with oversampling has stable superior performance in terms of AUC with different sizes of selected features. Especially, IEFS framework with oversampling works better than the other methods in terms of AUC metric with the top 30 selected features except the Thyroid data. Generally, the performance of IEFS framework using different filter feature selection methods does not show significant difference.

The computational cost of IEFS framework depends on the sampling preprocessing, the step *T*, and the number of classes. IEFS framework might consume more computational resources than the other filter feature selection methods, yet the extra effort for accuracy improvement is acceptable considering that the classification task is normally conducted offline.

## Conclusions

This paper proposes an iterative ensemble feature selection for imbalanced multiclass microarray data. The performance of conventional filter feature selection methods including Filter ranking, FCBF, and mRMR is compared to the IEFS framework on six gene microarray data sets. The results show that our proposed framework and OVA ensemble schema can obtain promising performance on multiclass gene selection problems. Within this framework different concrete oversampling methods can be applied for various multiclass gene selection problems. Undersampling does not work so well as oversampling in this framework due to the lack of training samples. In the future work, more effective oversampling methods beneficial to specific filter feature selection techniques will be developed and investigated with OVA classification schema. Moreover, the optimal combination of sampling method and feature selection will be explored. IEFS framework is also applicable to other domains suffering from the same problem.

## Methods

### The iterative ensemble feature selection

**Fig. 3:**
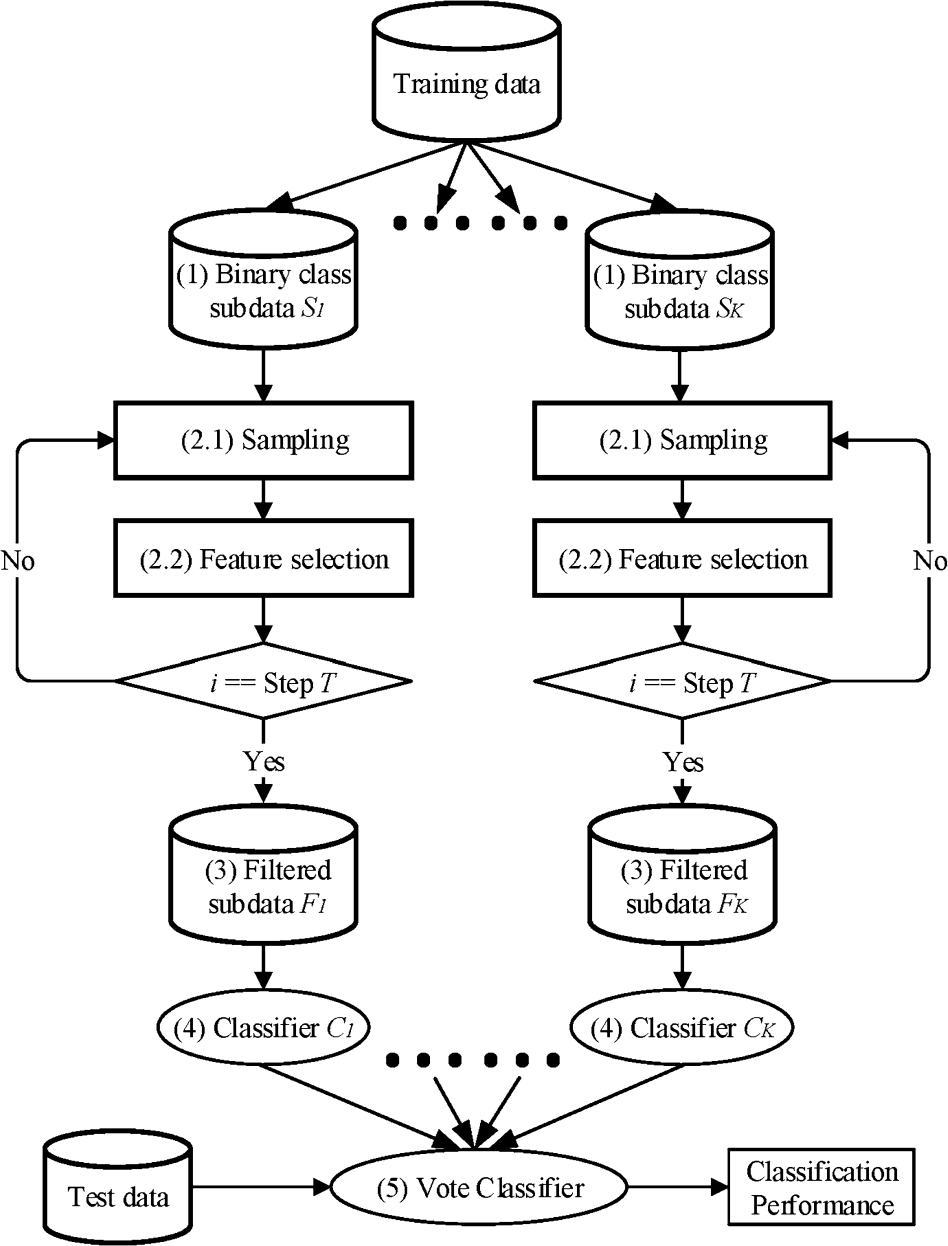
The iterative ensemble feature selection framework

In this section, we outline the IEFS framework for OVA classification in five stages as shown in Fig. 3. At the first step, a class binarization [13] transforms a *K*-class training data set into *K* binary class subdata sets (*S*_1_,…, *S*_K_) on the first stage [Fig. 3(1)]. At the second step, undersampling with sample decrease or oversampling with sample increase followed by feature selection is proceeded iteratively for *T* steps [Fig. 3(2.1, 2.2)]. The number of selected features decreases as the oversampling/undersampling carries on. The interval number *M*_*i*_ of decreasing or increasing samples in *i*th step is computed as:

1 $$M_{i} = \frac{{S_{M} - S_{m} }}{T}$$

where *S*_*M*_ denotes the number of samples in the majority class and *S*_*m*_ denotes the number of samples in the minority class in a binary classification. The interval size *N*_*i*_ of decreasing features in *i*th step is computed as:

2 $$N_{i} = \frac{{N_{M} - N_{n} }}{T}$$

where *N*_*M*_ denotes the number of all features and *N*_*n*_ denotes the target selected number. After data sampling and feature selection, the training data that is then filtered by getting rid of the unselected features [Fig. 3(3)]. The filtered training data *F*_*k*_ is used to train the binary classifier *C*_*k*_ at the fourth step [Fig. 3(4)]. Once each of the individual classifiers (*C*_1_,…, *C*_K_) is trained, the final prediction is made by taking the majority vote of the individual classifiers at the fifth step [Fig. 3(5)].

### Sampling methods

The random undersampling [26] and SMOTE oversampling [27] are used in the IEFS framework. The random undersampling method creates sample balance between the two classes by reducing the size of the majority one. This is accomplished by randomly removing samples from the majority class until the sizes of majority and minority classes are equal. The SMOTE algorithm generates new samples for the minority class. These samples are created artificially based on the feature space similarities between existing minority examples. By interpolating between the existing minority samples, a denser minority class containing more samples is achieved.

### Filter ranking feature selection

Filter ranking feature selection method first evaluates the univariate correlation between each feature and the class label vector based on mutual information and then ranks them in descending order. Afterward, a predefined number of top ranked features are selected. Filter ranking is widely used thanks to its easy implementation and high efficiency, but it cannot handle the redundancy between features.

### Fast correlation-based filter feature selection

FCBF [24] is a fast correlation-based filter feature selection method used in IEFS framework. It begins by ranking the features based on the correlation between features and the class label vector in a descending order and then removes those with correlation values smaller than a threshold *δ*. FCBF goes through the ranked feature list in decreasing order and a feature *f*_*i*_ is removed if there exist another feature *f*_*j*_ such that *SU*(*c*; *f*_*j*_) ≥ *SU*(*c*; *f*_*i*_) and *SU*(*f*_*i*_; *f*_*j*_) ≥ *SU*(*f*_*i*_; *c*) where *SU*(*a*; *b*) denotes the symmetrical uncertainty [34] between feature *a* and feature *b*. These two inequalities mean that *f*_*j*_ is better as a predicator of class label vector *c* and *f*_*i*_ is more similar to *f*_*j*_ than to *c*. The threshold *δ* can be adjusted to get the expected number of features.

### Minimum redundancy maximum relevance feature selection

The mRMR feature selection criterion was proposed by Peng et al. [25] to evaluate the candidate feature subset considering both feature redundancy and relevance at the meantime. Particularly, max-relevance, denoted as max D(*S*, *c*), refers to maximizing the relevance of a feature subset S to the class label *c*. In [25], the relevance of a feature subset is defined as:

3 $${\text{D}}\left( {{\text{S}},{\text{c}}} \right) = \frac{1}{\left| S \right|}\mathop \sum \limits_{{f_{i} \in S}}\Phi \left( {f_{i} ,c} \right)$$

where Φ(*f*_*i*_, *c*) denotes the relevance of a feature *f*_*i*_ to *c* based on mutual information.

If two relevant features highly depend on each other, the class-discriminative power would not change too much if one of them is removed. Feature redundancy is defined based on pair-wise feature dependence. Min-redundancy min *R*(*S*) is used to select a feature subset of mutually exclusively features. The redundancy of feature subset *R*(*S*) is defined as follows:

4 $${\text{D}}\left( {{\text{S}},{\text{c}}} \right) = \frac{1}{{\left| S \right|^{2} }}\mathop \sum \limits_{{f_{i} ,f_{j} \in S}}\Phi \left( {f_{i} ,f_{j} } \right)$$

mRMR is defined as the simple operator max Φ(*D*, *R*) = *D* − *R* which optimizes *D* and *R* simultaneously. In the experiment, linear forward search method [35] with window size 50 is used to find the near-optimal features. Given a feature subset S_*m*−1_ of *m* − 1 selected features, the task is to select the *m*-th feature that optimizes the following criterion:

$$\mathop {\hbox{max} }\limits_{{f_{j} \notin S_{m - 1} }} \left[ {{{\Phi }}\left( {f_{j} ,c} \right) - \frac{1}{m - 1}\mathop \sum \limits_{{f_{j} \in S_{m - 1} }}\Phi \left( {f_{i} ,f_{j} } \right)} \right]$$
